# *Antrodia camphorata* Mycelia Exert Anti-liver Cancer Effects and Inhibit STAT3 Signaling *in vitro* and *in vivo*

**DOI:** 10.3389/fphar.2018.01449

**Published:** 2018-12-17

**Authors:** Pei-Li Zhu, Xiu-Qiong Fu, Jun-Kui Li, Anfernee Kai-Wing Tse, Hui Guo, Cheng-Le Yin, Ji-Yao Chou, Ya-Ping Wang, Yu-Xi Liu, Ying-Jie Chen, Muhammad Jahangir Hossen, Yi Zhang, Si-Yuan Pan, Zong-Jie Zhao, Zhi-Ling Yu

**Affiliations:** ^1^Consun Chinese Medicines Research Centre for Renal Diseases, School of Chinese Medicine, Hong Kong Baptist University, Kowloon Tong, Hong Kong; ^2^Center for Cancer and Inflammation Research, School of Chinese Medicine, Hong Kong Baptist University, Kowloon Tong, Hong Kong; ^3^Research and Development Centre for Natural Health Products, HKBU Shenzhen Research Institute and Continuing Education, Shenzhen, China; ^4^Shenzhen Union Assets Biological Technology Co., Ltd., Shenzhen, China

**Keywords:** *Antrodia camphorata* mycelia, liver cancer, cell viability, apoptosis, metastasis, STAT3 signaling

## Abstract

Hepatocellular carcinoma (HCC), the major form of primary liver cancer, is a common cause of cancer-related death worldwide. Signal transducer and activator of transcription 3 (STAT3) signaling is constantly activated in HCC and has been proposed as a chemotherapeutic target for HCC. *Antrodia camphorata* (AC), a medicinal mushroom unique to Taiwan, is traditionally used for treating HCC. Whereas natural AC is scarce, cultured AC mycelia are becoming alternatives. In this study, we investigated the anti-HCC effects of the ethyl acetate fraction of an ethanolic extract of AC mycelia (EEAC), particularly exploring the involvement of STAT3 signaling in these effects. We found that EEAC reduced cell viability, induced apoptosis, and retarded migration and invasion in cultured HepG2 and SMMC-7721 cells. Immunoblotting results showed that EEAC downregulated protein levels of phosphorylated and total STAT3 and JAK2 (an upstream kinase of STAT3) in HCC cells. Real-time PCR analyses showed that STAT3, but not JAK2, mRNA levels were decreased by EEAC. EEAC also lowered the protein level of nuclear STAT3, decreased the transcriptional activity of STAT3, and downregulated protein levels of STAT3-targeted molecules, including anti-apoptotic proteins Bcl-xL and Bcl-2, and invasion-related proteins MMP-2 and MMP-9. Over-activation of STAT3 in HCC cells diminished the cytotoxic effects of EEAC. In SMMC-7721 cell-bearing mice, EEAC (100 mg/kg, i.g. for 18 days) significantly inhibited tumor growth. Consistent with our *in vitro* data, EEAC induced apoptosis and suppressed JAK2/STAT3 activation/phosphorylation in the tumors. Taken together, EEAC exerts anti-HCC effects both *in vitro* and *in vivo*; and inhibition of STAT3 signaling is, at least in part, responsible for these effects. We did not observe significant toxicity of EEAC in normal human liver-derived cells, nude mice and rats. Our results provide a pharmacological basis for developing EEAC as a safe and effective agent for HCC management.

## Introduction

Hepatocellular carcinoma (HCC) is the predominant form of liver cancer, accounting for 70–85% of primary liver cancer cases worldwide. It is the fifth most common cancer and the second leading cause of cancer-related death globally (Torre et al., 2015). China accounts for about 55% of HCC cases in the world (Song et al., 2013). HCC is known as a fatal cancer because it is typically not diagnosed until the cancer reaches an advanced stage, and because there are no effective drugs, particularly for such late stages. The mean survival time after diagnosis is less than a year (Somboon et al., 2014; Intaraprasong et al., 2016; Wanich et al., 2016). Conventional chemotherapeutic drugs (e.g., doxorubicin and cisplatin) and targeted therapies (e.g., sorafenib and brivanib) show modest survival benefits in HCC patients; but most of these treatments exhibit significant side effects (Rajendran et al., 2011).

STAT3 is commonly activated in a variety of human cancers, including HCC (He et al., 2010). Activated STAT3 is found in approximately 60% of human HCC specimens. Recent studies indicate that constitutive activation/phosphorylation of STAT3 promotes HCC cell proliferation, survival, angiogenesis, immune evasion, metastasis (Sun et al., 2015; Cacalano, 2016), and chemo-resistance (Ma et al., 2016). Blocking STAT3 activation is able to inhibit HCC growth and metastasis (Sui et al., 2014; Cheng et al., 2017; Wu et al., 2017). These reports suggest that STAT3 plays a role in HCC progression, and is a potential target for HCC therapy. Although some STAT3 inhibitors, such as NSC 74859 (Lin et al., 2009) and LLL12 (Zuo et al., 2015), have been reported to inhibit STAT3 activation in HCC cells, no STAT3 inhibitor has been approved for treating HCC.

*Antrodia camphorata* (AC) is a unique type of fungus which grows on the heartwood wall of the endemic tree species *Cinnamomum kanehirai* Hay (Lauraceae) in Taiwan. AC has been traditionally used as a remedy for liver diseases. Pharmacological studies indicate that AC possesses health-related beneficial effects, such as hepatoprotective, anti-inflammatory, immunomodulatory, anti-hypertensive and anticancer activities (Geethangili and Tzeng, 2011). Crude extracts or constituents of AC have been reported to have anti-liver cancer properties (Hsu et al., 2005; Hsieh et al., 2011; Lai et al., 2016). Several signaling pathways, such as the PI3K/AKT, AMPK/mTOR and JAK2/STAT3 pathways, are shown to be involved in these effects (Chiang et al., 2010; Chen et al., 2014, 2015). Because the tree species in the wild is becoming endangered, mass production of AC in nature is difficult. To solve this problem, cultured AC mycelia are used as alternatives. Cultured AC mycelia have been shown to exert anti-cancer effects against a variety of cancer types including liver cancer (Song et al., 2005a). However, the mechanism of action of AC mycelia remains largely unknown. Previous studies demonstrated that the ethyl acetate fraction of a methanolic extract from AC induced apoptosis in HCC cells (Hsu et al., 2005). In addition, the ethyl acetate fraction showed the strongest inhibitory activity against colon cancer cell proliferation among different fractions (Park et al., 2013). In this study, we investigated the anti-HCC effects of the ethyl acetate fraction of an ethanolic extract of AC mycelia (EEAC for short), and the involvement of STAT3 signaling in these effects. We also evaluated the safety of EEAC in animals.

## Materials and Methods

### Reagents and Antibodies

3-(4, 5-dimethylthiazol-2-yl)-2, 5-diphenyltetrazolium bromide (MTT) was bought from Sigma. Annexin V/PI binding kit was purchased from Abcam (Cambridge, MA, United States). Antibodies against p-STAT3 (Tyr705), STAT3, p-JAK2 (Tyr1007/1008), JAK2, PARP, cleaved caspase-3, -8, -9, Bcl-xL, Bcl-2, MMP-2 and MMP-9 were purchased from Cell Signaling Technology (Beverly, MA, United States). Antibodies against GAPDH and SP1 were purchased from Santa Cruz Biotechnology (Santa Cruz, CA, United States), goat anti-rabbit IgG, goat anti-mouse IgG and protein markers were supplied by Bio-Rad (Hercules, CA, United States). All materials used for cell culture were purchased from Life Technologies Inc. (GIBCO, United States). Assay kits for serum biochemical parameters were purchased from Nanjing Jiancheng Bioengineering Institute (Nanjing, China).

### Cultivation of AC Mycelia

Mycelia of *Antrodia camphorata* were cultured by Hong Kong Chinese Medical Science Academy Ltd. (He et al., 2012). Briefly, *Antrodia camphorata* was bought from the American Type Culture collection (ATCC, United States). Stocked mycelia were transferred into a 250 ml Erlenmeyer flask with 100 ml seed medium (including glucose 20.0 g/l, malt extract 20.0 g/l and peptone 1.0 g/l, with initial pH 5.0) and incubated at 25°C for 7 days. These mycelia were used as inoculum for subsequent experiments. The inoculum was then cultured in a liquid medium consisting of 20.0 g/l corn starch, 20.0 g/l wheat bran, 1.85 g/l MgSO_4_; pH was adjust to 3.0 for 16 days before harvest. Contents of heavy metals and aflatoxins in AC mycelia were detected according to National Standard of the People’s Republic of China for determination of As, Pb, Hg, Cd and aflatoxins in foods. Cd, Hg and aflatoxins were not detected in the mycelia (Determination of heavy metals and aflatoxins are described in the Supplementary Material).

### Preparation of EEAC

Powdered mycelia were extracted 3 times with 95% ethanol for 1 h each under reflux and then centrifuged at 4000 × *g* for 5 min. After removing the precipitate, the supernatant was sequentially partitioned between water and ethyl acetate (EA) (1:3, v/v) three times to give an EA fraction. The obtained extract was then lyophilized with a Virtis Freeze Dryer (Virtis Company, New York, NY, United States) to obtain EEAC powder (EEAC for short, yield: 22.17%). UPLC analyses showed that contents of (25S)-Antcin H and (25R)-Antcin A, triterpenoids occurring in AC, in EEAC are 3.87% and 0.08%, respectively (Supplementary Figure S1).

### Cell Culture

Human HCC cells (PLC/PRF/5 and HepG2) and normal human liver-derived cells (MIHA) were purchased from American Type Culture Collection (ATCC, United States). Human HCC cells (SMMC-7721, Huh7, MHCC 97, MHCC 97L, HCCLM3) were obtained from Cell Bank of Type Culture Collection of Chinese Academy of Sciences (Shanghai, China). All cells were cultured in Dulbecco’s modified Eagle’s medium (DMEM) supplemented with 10% heat-inactivated fetal bovine serum (FBS) and 1% penicillin/streptomycin (P/S). The cells were maintained at 37°C in a humidified incubator with 5% CO_2_.

### MTT Assay

The cytotoxic effects of EEAC on PLC/PRF/5, HepG2, SMMC-7721, Huh7, MHCC 97, MHCC 97L, HCCLM3 and MIHA cells were determined by MTT assay. Cells (4,000/well) were incubated on 96-well plates in the presence or absence of EEAC (50, 100, 200, 400 μg/ml) for 24 or 48 h. Thereafter, 10 μl of MTT solution (5 mg/ml) was added to each well. After 2-h incubation, 100 μl of DMSO was added to dissolve the formed formazan crystal. The optical density (OD) was measured at 570 nm by a microplate spectrophotometer (BD Biosciences, United States) (Fu et al., 2018).

### Annexin V/PI Assay

Apoptosis in HCC cells was detected using Annexin V/PI binding kit according to manufacturer’s introductions. Briefly, 2 × 10^5^ HepG2 and SMMC-7721 cells were seeded on 6-well plates and allowed to adhere overnight. After a 48-h treatment with vehicle or EEAC (60, 90, 120 μg/ml), cells were harvested and re-suspended in labeling solution (500 μl of 1X binding buffer, 5 μl of AnnexinV-FITC and 5 μl of PI) in the dark at room temperature for 5 min. After that, flow cytometric analyses were performed on a FACSCalibur^TM^ platform (BD Biosciences, United States) utilizing 10,000 events (Tse et al., 2014).

### Cell Migration and Invasion Assays

HepG2 and SMMC-7721 cells were cultured in serum-containing media on six-well plates until about 70% confluence was achieved. The medium was replaced by serum-free medium after three washes with PBS and incubated for 24 h. Then cells were treated with EEAC (30, 60 μg/ml) in serum-free medium for 24 h. The cells were trypsinized, counted and re-suspended in serum-free medium. For the transwell migration assay, 4 × 10^4^ cells were placed into the upper chamber of each cell culture insert with a non-coated membrane (BD Biosciences, San Jose, CA, United States). For the invasion assay, 4 × 10^4^ cells were placed into the upper chamber of each matrigel-coated insert. In both assays, 0.70 ml of serum-containing medium was placed in the lower chamber. After incubation at 37°C for 48 h, cells and matrigel remaining in the upper chamber were removed by scrubbing with a cotton swab. Migrated or invaded cells were fixed with 100% methanol and then stained using 0.1% crystal violet solution. Cells in four random visual fields under a light microscope were counted and photographed (Cao et al., 2016).

### Subcellular Fractionation

HepG2 cells (5 × 10^5^ cells/well) were seeded in 60-mm dishes, and then treated with either vehicle or EEAC (60, 90, 120 μg/ml) for 24 h. Then cells were collected and re-suspended in 400 μl of hypotonic lysis buffer and incubated on ice for 15 min. Thereafter, 12 μl of Nonidet P-40 (NP-40) (10%, v/v) was added, and cells were kept on ice for another 10 min. After centrifugation, supernatants were collected as the cytoplasmic extracts. The nuclei pellets were then incubated with high salt buffer on ice for 30 min. After centrifugation, the lysates were taken as nuclear fractions (Li et al., 2017).

### Western Blot Analysis

Protein samples, prepared from cultured cells or tumor tissues, were analyzed by Western blot analyses as described previously (Cao et al., 2014). The membranes were incubated with primary antibodies at 4°C overnight. After 3 washes with TBS-T, membranes were re-incubated with HRP-conjugated secondary antibodies for 1 h at room temperature. Immunoreactive bands were visualized by chemiluminescence (ECL, Invitrogen, United States). The intensity of the immunoreactive bands was analyzed and quantified using Image J software.

### Plasmids Transfection and Luciferase Reporter Assay

Constitutively active STAT3 construct STAT3-C Flag pRc/CMV and STAT3 reporter construct 4xM67 pTATA TK-Lu were purchased from Addgene (United States). Transfection of STAT3C plasmids into HepG2 cells was performed as described previously (Su et al., 2017). Cells were transfected with plasmids for 48 h before functional assays were carried out. For luciferase reporter assay, cells were co-transfected with STAT3 reporter construct 4xM67 pTATA TK-Lu plus *Renilla* luciferase expression vector PRL-CMV (Promega, United States) using the lipofectamine 2000 reagent (Invitrogen, United States). After 24-h transfection, cells were treated with vehicle or EEAC (60, 90, 120 μg/ml) for another 24 h. Firefly and renilla luciferase activities were detected using the dual-luciferase reporter assay system (Promega, United States). Firefly luciferase activity was normalized to renilla luciferase activity in cell lysate as described elsewhere (Tse et al., 2017).

### Quantitative Real-Time Polymerase Chain Reaction (qRT-PCR)

Total RNA was extracted with Trizol reagent (Invitrogen, United States) and reverse-transcripted with oligo-dT using M-MLV reverse transcriptase (Promega, United States) according to the manufacturer’s protocol. Gene expression levels were determined by qRT-PCR analyses using SYBR green reaction mixture in the ViiA 7 Real Time PCR System (Applied Biosystems, United States) (Su et al., 2013). Data were analyzed by the ΔΔCt method and normalized to GAPDH. Primer sequences were list as follows: human STAT3: forward, GGCCCAATGGAATCAGCTACAG, reverse, GAAGAAACTGCTTGATTCTTCG; Human JAK2: forward, TCTGGGGAGTATGTTGCAGAA, reverse, AGACATGGTTGGGTGGATACC; Human GAPDH: forward, ATCATCAAGCAATGCCTCCTG, reverse, ATGGACTGTGGTCATGAGTC.

### *In vivo* Studies

All care and handling of animals were performed with the approval of the Department of Health, Hong Kong. Experimental procedures were approved by the Committee on the Use of Human & Animal Subjects of the Hong Kong Baptist University [(16-19) DH/HA & P/8/2/6 Pt. 5].

Sprague-Dawley (SD) rats (5 weeks old) were purchased from the Laboratory Animal Service Centre, Chinese University of Hong Kong. No minimum lethal dose was found. Thus, maximum tolerable dose (MTD) of rats was determined in acute oral toxicity study. Repeated dose 28-day oral toxicity was assessed in SD rats as well. Detailed protocols for safety assessments are described in the Supplementary Material.

Male Balb/c nude mice (6–8 weeks old) were purchased from BioLASCO Taiwan (Taipei, Taiwan). SMMC-7721 cells (3 × 10^6^ cells in 0.1 ml PBS) were injected subcutaneously into the backs of nude mice. Immediately after cell injection, mice were randomly assigned to three groups (*n* = 6 for each group) and then intragastrically (i.g.) administrated with 0.4 ml vehicle solution containing 30% PEG-40, 5% Tween-80 and 4% DMSO, 50 mg/kg EEAC or 100 mg/kg EEAC daily for 18 consecutive days. Tumor volume was recorded every 3 days using Vernier calipers and calculated according to the following formula: (width^2^ × length)/2. At the end of the experiment, tumor xenografts were dissected and weighted. Parts of the tumor tissues were fixed in formalin for TUNEL assays, and parts of them were stored at -80°C for Western blotting.

### Fluorometric TUNEL System

In tumor tissues, apoptotic cells were determined using the Fluorometric TUNEL System as described previously (Zhang et al., 2013). Cell nuclei with green fluorescent staining were defined as TUNEL-positive nuclei. TUNEL-positive cells in 5 random fields on each section at 200× magnification were counted.

### Serum Biochemical Analysis

At the end of the study, blood samples of nude mice were collected. Serum ALT, AST and ALP levels were determined according to the manufacturer’s instruction.

### Statistical Analysis

Statistical analysis was performed using GraphPad Prism version 5.0 (GraphPad software, San Diego, CA, United States). Data from cellular and animal assays were presented as mean ± SD and mean ± SEM, respectively. Statistical significance was determined by one-way ANOVA followed by the Dunnett’s multiple comparisons. *P* < 0.05 was considered statistically significant.

## Results

### EEAC Reduces Viability and Induces Apoptosis in Human HCC Cells

**FIGURE 1:**
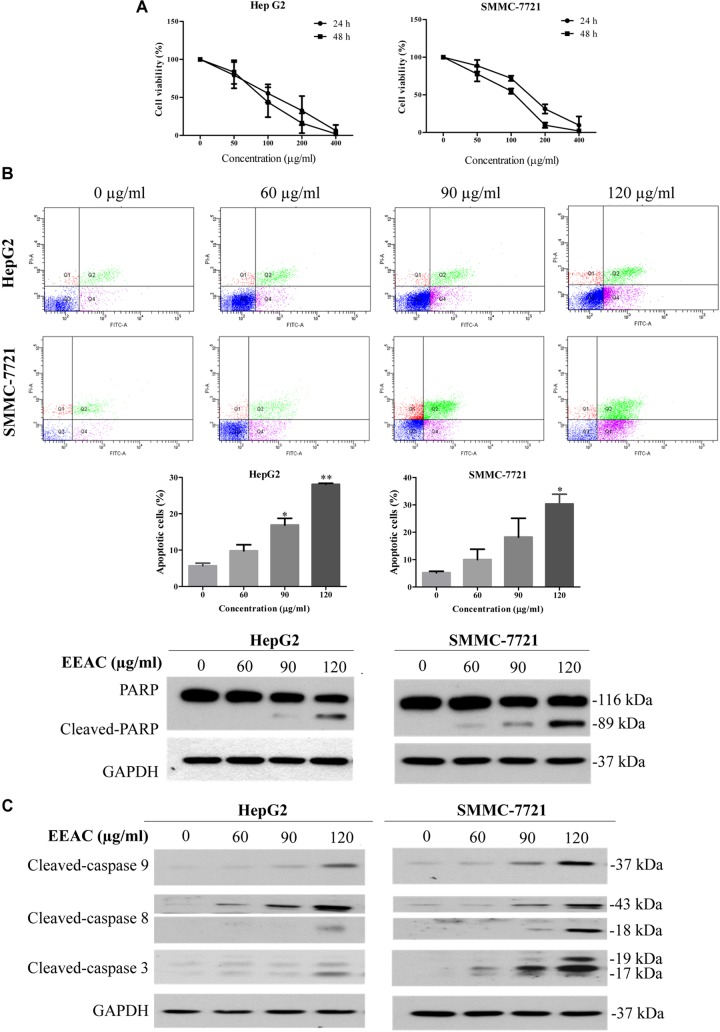
EEAC reduces viability and induces apoptosis in human HCC cells. **(A)** Effects of EEAC on cell viability in HpeG2 and SMMC-7721 cells. Cells were treated with various concentrations of EEAC for 24 and 48 h, respectively. Cell viability was assessed by MTT assays. **(B)** Effects of EEAC on apoptosis in HCC cells. Cells were exposed to EEAC at indicated concentrations for 48 h and percentage of apoptotic cells was determined by flow cytometry after Annexin V/PI double staining. Data were shown as mean ± SD of three independent experiments. ^∗^*P* < 0.05, ^∗∗^*P* < 0.01 vs. control. Protein levels of PARP and cleaved-PARP in HCC cells were explored by western blotting following EEAC treatment for 24 h. **(C)** Western blot analyses for protein levels of cleaved-caspase-9, -8, and -3 in HCC cells. Results shown are representative of three independent experiments.

To evaluate the anti-HCC effects of EEAC *in vitro*, MTT assays were performed on a panel of human HCC cells. Results showed that HepG2 and SMMC-7721 were the most sensitive to EEAC treatment among the tested cell lines. Moreover, EEAC exhibited lower cytotoxicity to normal human liver-derived cells (MIHA) than to HCC cells (Supplementary Figure S2). HepG2 and SMMC-7721 cells were chosen for subsequent investigations. As shown in Figure 1A, EEAC reduced viabilities of HepG2 and SMMC-7721 cells in dose- and time-dependent manners, with IC_50_ values of 107.3 ± 22.2 and 142.7 ± 9.3 μg/ml, respectively, for the 24-h treatment. To investigate the possibility that EEAC triggers apoptosis in human HCC cells, Annexin V/PI double staining assay was conducted. Flow cytometric analyses demonstrated that the percentage of apoptotic cells in EEAC-treated HepG2 and SMMC-7721 cells were significantly increased when compared with vehicle-treated cells. The cleavage of poly (ADP-ribose) polymerase (PARP) in EEAC-treated HCC cells further validated the pro-apoptotic effects of EEAC (Figure 1B). Western blotting results showed that EEAC up-regulated protein levels of cleaved caspase-9, cleaved caspase-8, and cleaved caspase-3 in HCC cells (Figure 1C).

### EEAC Dampens HCC Cell Migration and Invasion

**FIGURE 2:**
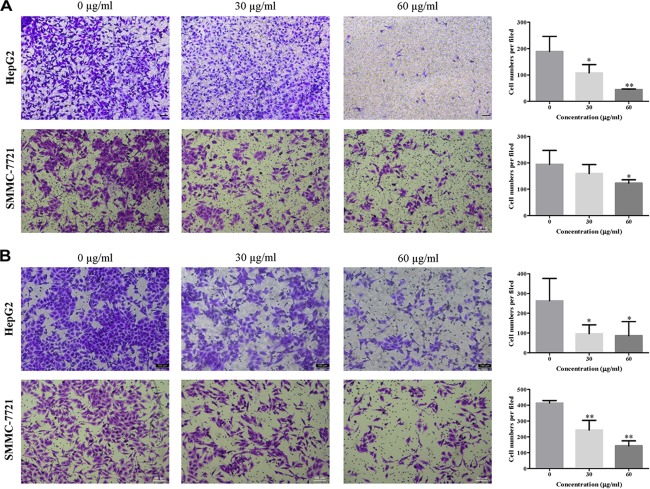
EEAC dampens HCC cell migration and invasion. **(A)** migration assays and **(B)** invasion assays were performed in HepG2 and SMMC-7721 cells for 48 h after pre-treatment with indicated concentrations of EEAC for 24 h. Representative images are shown on the left and the quantification of 4 randomly selected fields was shown on the right. Scale bar = 100 μm. Data were shown as mean ± SD of three independent experiments. ^∗^*P* < 0.05, ^∗∗^*P* < 0.01 vs. control.

To explore the effects of EEAC on HCC cell migratory and invasive abilities, transwell migration and invasion assays were performed, respectively. As shown in Figures 2A,B, the number of cells transferred to the lower membrane of the chamber in EEAC-treated groups was fewer than that in vehicle-treated groups in HepG2 and SMMC-7721 cells.

### EEAC Inhibits Activation and Lowers mRNA Level of STAT3 in Human HCC Cells

**FIGURE 3:**
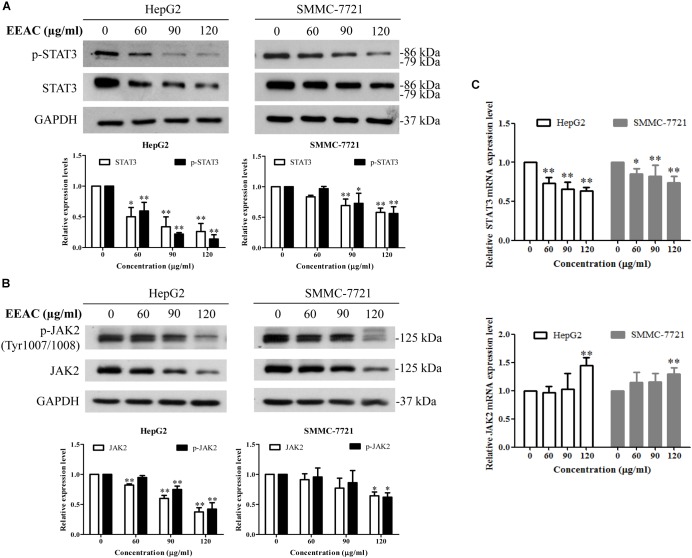
EEAC inhibits activation and lowers mRNA level of STAT3 in human HCC cells. Cells were treated with indicated concentrations of EEAC for 24 h, and then protein levels of **(A)** p-STAT3 and STAT3 and **(B)** p-JAK2 and JAK2 were determined by Western blot analyses. The relative protein levels were analyzed by Image J software. **(C)** mRNA levels of STAT3 (upper panel) and JAK2 (lower panel) in HepG2 and SMMC-7721 cells were detected by qRT-PCR. Data were shown as mean ± SD of three independent experiments. ^∗^*P* < 0.05, ^∗∗^*P* < 0.01 vs. the corresponding control.

To determine the effects of EEAC on the activation/phosphorylation of STAT3 in human HCC cells, immunoblotting assay was performed. As shown in Figure 3A, EEAC dose-dependently suppressed the phosphorylation of STAT3 at the Tyr705 site and lowered protein levels of total STAT3 in both HepG2 and SMMC-7721 cells. We also found that EEAC lowered protein levels of p-JAK2 (at Tyr1007/1008, JAK2 is an upstream tyrosine kinase of STAT3) and total JAK2 (Figure 3B). To investigate whether the reduction of protein levels of STAT3 and JAK2 are due to the inhibition of gene expressions, qRT-PCR analyses were performed. As shown in Figure 3C, STAT3, but not JAK2, mRNA levels were down-regulated by EEAC in a dose-dependent manner. These results indicate that EEAC not only inhibits the activation of STAT3 but also negatively regulates the gene expression of STAT3 in HCC cells.

### EEAC Reduces STAT3 Nuclear Pool and Suppresses STAT3-Luciferase Reporter Activity in Human HCC Cells

**FIGURE 4:**
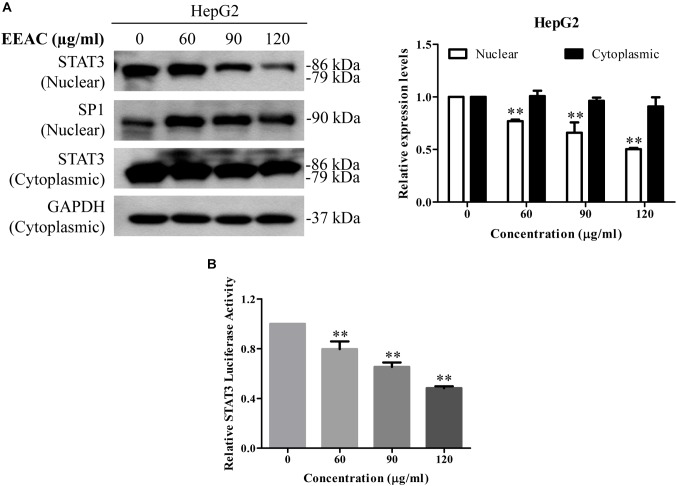
EEAC reduces STAT3 nuclear pool and suppresses STAT3-luciferase reporter activity in human HCC cells. **(A)** Protein levels of STAT3 in cytoplasmic and nuclear extracts. HepG2 cells were treated with indicated concentrations of EEAC for 24 h. Protein levels of STAT3 were determined by Western blot analyses (left) and relative expression levels were analyzed by Image J software (right). GAPDH and SP1 were served as loading controls of cytoplasmic and nuclear extractions, respectively. **(B)** HepG2 cells were transfected with STAT3-luciferase reporter plasmid with a PRL-CMV construct for 48 h, and then treated with EEAC at indicated concentrations for another 24 h. Transcriptional activity of STAT3 was measured by the dual-luciferase reporter assay. Data were shown as mean ± SD of three independent experiments. ^∗∗^*P* < 0.01 vs. the control.

As nuclear translocation is critical to the function of transcription factors, we then determined whether EEAC affects the nuclear protein level of STAT3 in HCC cells. Western blotting results demonstrated that treatment with EEAC significantly lowered the nuclear protein level of STAT3 in HepG2 cells (Figure 4A). Suppression of STAT3 nuclear localization will sequentially impede gene transcriptional activity of STAT3 (Cao et al., 2016). We, therefore, examined whether EEAC affected STAT3 transcriptional activity in HepG2 cells. As shown in the luciferase assay (Figure 4B), STAT3-luciferase reporter activity was suppressed by EEAC treatment in a dose dependent manner.

### EEAC Downregulates Protein Levels of STAT3-Targeted Molecules

**FIGURE 5:**
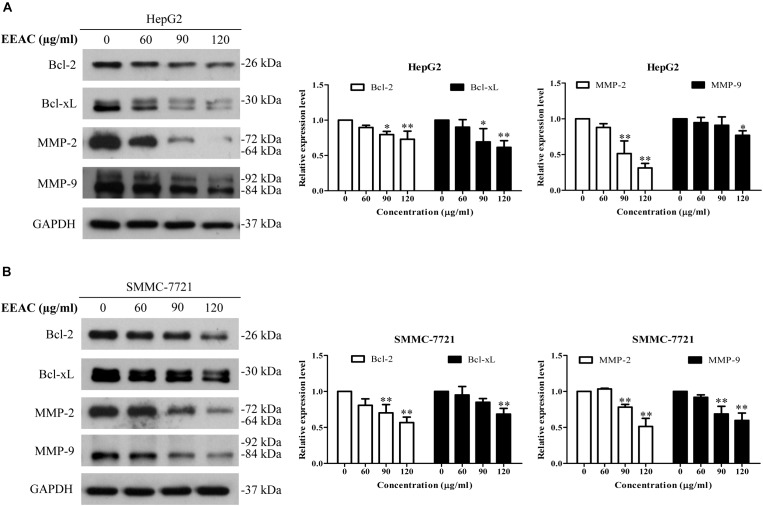
EEAC downregulates protein levels of STAT3-targeted molecules. HepG2 **(A)** and SMMC-7721 **(B)** cells were treated with indicated concentrations of EEAC for 24 h, and then protein levels of Bcl-2, Bcl-xL, MMP-2 and MMP-9 were determined by immunoblotting. The relative protein levels were analyzed by Image J software and shown as mean ± SD of three independent experiments.^∗^*P* < 0.05, ^∗∗^*P* < 0.01 vs. the corresponding control.

Western blotting assay was employed to determine the effects of EEAC on protein levels of STAT3-targeted molecules. We found that EEAC decreased protein levels of STAT3-trageted molecules, including Bcl-2, Bcl-xL, MMP-2 and MMP-9, in both HepG2 and SMMC-7721 cells (Figure 5).

### Over-Activation of STAT3 in HepG2 Cells Diminishes the Cytotoxic Effects of EEAC

**FIGURE 6:**
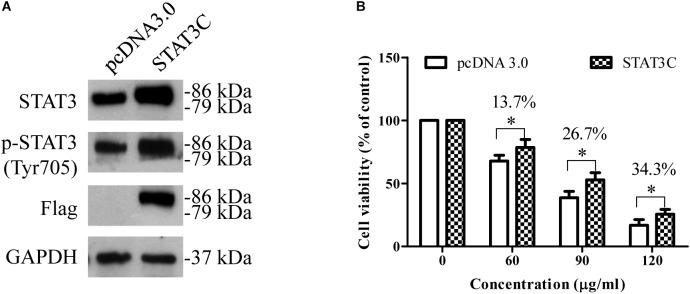
Over-activation of STAT3 in HepG2 cells diminishes the cytotoxic effects of EEAC. **(A)** Protein levels of STAT3 and p-STAT3. HepG2 cells were transiently transfected with either an empty vector or an STAT3C-expressing construct for 48 h, and then total cell lysates were extracted for Western blot analyses. **(B)** After transfection, HepG2 cells were treated with EEAC for 48 h, the cell viability was determined by the MTT assay. Data were shown as mean ± SD of three independent experiments. ^∗^*P* < 0.05 vs. cells transfected with the empty vector.

To further determine the role of STAT3 inhibition in the anti-HCC effects of EEAC, HepG2 cells were transiently transfected with constitutively active STAT3 (STAT3C) plasmid for 48 h, and then treated with EEAC for 24 h. The effect of EEAC on cell viability was determined by the MTT assay. As shown in Figure 6A, transient transfection of STAT3C evidently elevated protein levels of STAT3 and p-STAT3 (Try705) when compared with the empty vector transfection. MTT assays showed that the cytotoxic effects of EEAC were diminished by STAT3 over-activation in HepG2 cells (Figure 6B).

### EEAC Suppresses Tumor Growth in SMMC-7721 Cell-Bearing Mice

**FIGURE 7:**
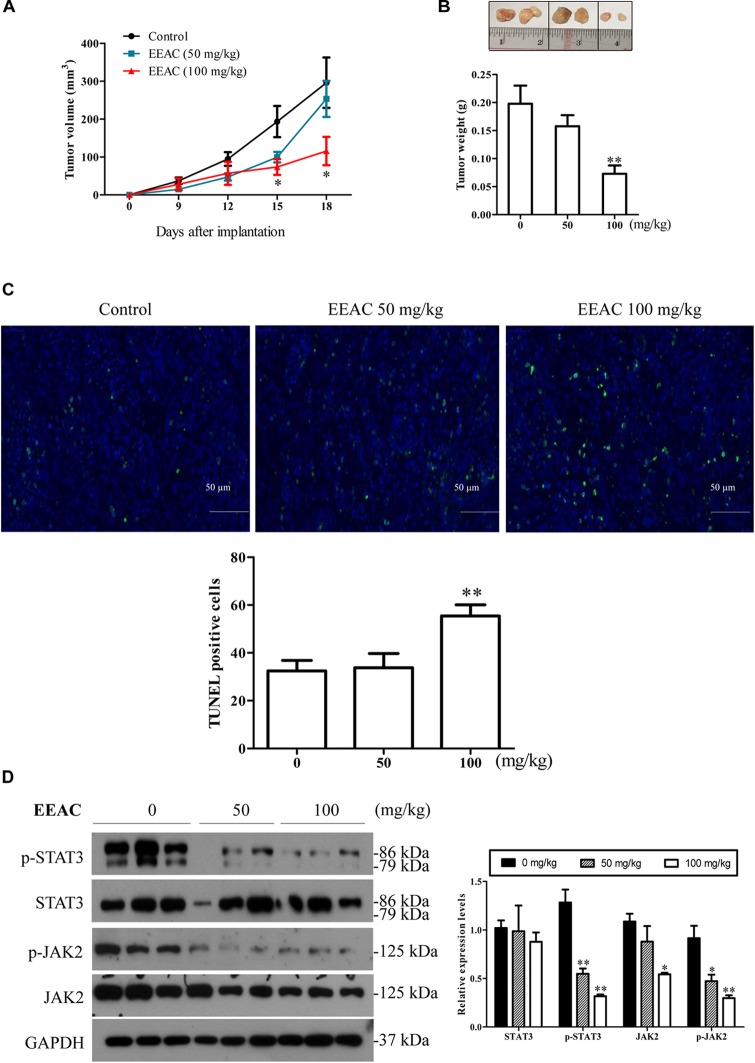
EEAC suppresses tumor growth in SMMC-7721 cell-bearing mice. Nude mice bearing SMMC-7721 xenograft were treated with EEAC for 18 days. **(A)** Tumor volume. **(B)** Representative tumors removed from mice. Tumor weights were recorded. In **(A,B)**, data were presented as mean ± SEM, *n* = 6. **(C)** TUNEL assays for apoptosis in tumor tissues collected from three individual mice. **(D)** Western blot analyses for protein levels of p-STAT3, STAT3, p-JAK2 and JAK2 (left panel) and the relative protein levels were analyzed by Image J software (right panel). Data were shown as mean ± SEM, *n* = 3. ^∗^*P* < 0.05, ^∗∗^*P* < 0.01 vs. vehicle control.

To investigate the anti-HCC effects of EEAC *in vivo*, a SMMC-7721 xenograft Balb/c nude mouse model was used. Mice were daily intragastrically administrated with different doses of EEAC or vehicle for 18 days. At the end of the treatment, tumor weight was recorded. During the experimental period, all mice developed visible subcutaneous tumors on day 7. There was no significant difference in tumor volumes between vehicle control group and EEAC-treated groups on day 9; however, tumor volumes in EEAC-treated groups were significantly smaller than that in the control group after 15 days (Figure 7A). The average tumor weights in 50 mg/kg and 100 mg/kg EEAC-treated groups were 79.7 and 36.8%, respectively, of that of the control group (Figure 7B). No animal died during the experimental period. No significant differences were found in body weight gain and food consumption. Behavioral and physical examinations did not show any signs of toxic effects. No abnormality was observed at the gross necropsy for pivotal organs, including liver, kidney, heart and stomach (Data not shown). EEAC did not affect mouse serum ALT, AST and ALP levels as well (Table 1). Consistent with the results in HCC cells, EEAC induced apoptosis in tumor tissues, which was detected by TUNEL assays. The apoptotic cells (with green nuclei) in tumors of EEAC-treated mice were more than that in tumors of vehicle-treated mice (Figure 7C). EEAC also lowered protein levels of p-STAT3 (Tyr705), p-JAK2 (Tyr1007/1008), and JAK2, but not STAT3, in tumors (Figure 7D). In summary, we found that EEAC at 100 mg/kg significantly inhibits tumor growth in SMMC-7721 cell-bearing mice, as well as induces apoptosis and suppresses STAT3 activation in tumors.

**Table 1:** Effects of EEAC on serum biochemical parameters in SMMC-7721 cell-bearing mice (mean ± SEM, *n* = 6).

Dose (mg/kg)	ALT (U/L)	AST (U/L)	ALP (King unit/100 ml)
0	9.93 ± 1.59	13.02 ± 1.04	9.15 ± 0.56
50	8.37 ± 1.19	12.40 ± 0.78	8.40 ± 0.39
100	6.72 ± 0.92	10.69 ± 0.67	9.35 ± 2.02


## Discussion

Accumulating evidence suggests that certain mushrooms are highly valuable for their nutritional and pharmacological properties, including anti-cancer activities (Cizmarikova, 2017; Zeng and Zhu, 2018). AC is one such mushroom, which has been shown to have anti-cancer effects (Peng et al., 2006; Hseu et al., 2008; Chiou et al., 2011; Yang et al., 2011; Yang et al., 2014). In this study, we demonstrated that EEAC exerted anti-HCC effects by reducing cell viability and inducing apoptosis in HCC cells. Song et al. (2005b) reported that the methanol extract of AC mycelia induced apoptosis in cultured cells *via* activating the activities of caspase-8 and caspase-3. Our results showed that both the extrinsic and intrinsic apoptotic pathways were involved in EEAC-induced apoptosis in HCC cells, as evidenced by the appearances of cleaved caspase-8 (an indicator of activation of the extrinsic pathway), -9 (an indicator of activation of the intrinsic pathway) and -3. Triterpenoids have been reported to be the anticancer components of AC (Yeh et al., 2009; Lee et al., 2012). In our study, we found that the IC_50_ value of (25S)-Antcin H, a triterpenoid compound identified in EEAC, was higher than 200 μM (Supplementary Figure S3), which is comparable to findings in other cell lines (Chiu et al., 2017). Since (25S)-Antcin H poses low cytocoxicity to liver cancer cells, we hypothesize that it is not primarily responsible for the anti-proliferative effects of EEAC.

Subcutaneous xenograft mouse model is the most commonly used implantation model in the study of HCC (He et al., 2015). In this study, we found that EEAC exerted potent prophylatic effects on tumor growth in SMMC-7721 cell-bearing mice. No treatment-related toxicity was observed in mice during the experimental period. We also evaluated the safety of EEAC in rats. No death or treatment-related signs of toxicity were observed in SD rats receiving EEAC at a single dose of 5000 mg/kg body weight in an acute oral toxicity study (Supplementary Table S2). The no-observed-adverse-effect level (NOAEL) for repeated dose 28-day oral toxicity study was higher than 1000 mg/kg BW (Supplementary Figure S4 and Supplementary Table S3–S5). Comprehensive safety assessment of EEAC is warranted; however, we have found no evidence that EEAC is not safe. Although some of the active components in AC have been shown to have anti-HCC effects, no AC-derived anti-HCC agent is available. In the future, we hope to develop EEAC and/or its active compounds into anti-HCC agents.

STAT3 is a cytoplasmic transcription factor that transmits signals from the plasma membrane to target genes in the nucleus. The activation of STAT3 at Tyr705 is linked to malignant cancer behaviors, including cell proliferation, migration, invasion and metastasis. Therefore, inhibition of STAT3 activation represents a potential therapeutic approach for human cancers. In this study, we found that EEAC suppressed the phosphorylation of STAT3 at Tyr705 in both human HCC cells and tumors. Over-activation of STAT3 diminished the cytotoxic effects of EEAC in HepG2 cells, indicating that inhibition of STAT3 is involved in the anti-HCC effects of EEAC. In HCC cells, activation/phosphorylation of STAT3 at tyrosine 705 residue is majorly mediated by JAK family kinases, especially JAK2 (Fang et al., 2017). Compounds identified from AC have been reported to inhibit the phosphorylation of JAK2 and STAT3 in cancer cells (Yeh et al., 2013; Chen et al., 2014). Reduction of p-STAT3 (Tyr705) can probably be attributed to the suppression of JAK2 activation. Indeed, we found that EEAC markedly lowered protein levels of p-JAK2 (Tyr1007/1008) both *in vivo* and *in vitro*. Moreover, EEAC lowered protein levels of JAK2 in HCC cells without significantly affecting JAK2 mRNA levels, suggesting that EEAC suppressed JAK2 protein expression at the post-transcriptional level. JAK2 proteins can be degraded through the ubiquitin-proteasome pathway (Ungureanu et al., 2002; Hatakeyama, 2012). Whether ubiquitin-mediated degradation is responsible for EEAC-induced reduction of total JAK2 needs to be further studied. In HCC cells, protein levels of total STAT3 were decreased by EEAC treatment as well, which may be because of the observed reduction of STAT3 mRNA expression. Downregulation of STAT3 mRNA expression has been reported to suppress the proliferation and migration of tumor cells (Shen et al., 2016). Suppressing STAT3 mRNA expression also enhances the sensitivity of HCC cells to chemotherapies (Xue et al., 2016). The observed anti-HCC activities in our *in vitro* and *in vivo* models may be because of, at least in part, the inhibitory effects of EEAC on STAT3 activation and gene expression.

When STAT3 becomes phosphorylated at tyrosine-705 (Tyr705), it forms a homodimer and then undergoes nuclear translocation. In cell nucleus, STAT3 regulates the expression of a specific set of genes that encode for cell survial-related proteins and metastasis-related proteins (Carpenter and Lo, 2014). We, therefore, speculated that inhibition of STAT3 phosphorylation might reduce the nuclear translocation and transcriptional activity of STAT3. As expected, we found that treatment with EEAC decreased the nuclear protein level and luciferase reporter activity of STAT3 in HCC cells. We further found that EEAC decreased protein levels of STAT3-trageted molecules Bcl-2 and Bcl-xL in HCC cells. Bcl-2 and Bcl-xL are anti-apoptotic proteins whose overexpression can inhibit apoptosis and confer chemoresistance in HCC cells (Chun and Lee, 2004; Yang et al., 2014). The promoters of both Bcl-2 and Bcl-xL contain STAT3 binding sites and can be upregulated by STAT3 in cancer cells (Carpenter and Lo, 2014). Therefore, EEAC-induced apoptosis in HCC cells might be related to STAT3 inhibition-mediated down-regulation of Bcl-2 and Bcl-xL. Matastasis-related proteins MMP-2 and MMP-9 are also STAT3 targets (Carpenter and Lo, 2014). Activation of STAT3 has been reported to promote matestasis *via* regulating the production of MMP-2 and MMP-9 in a variety of tumors (Ko et al., 2016; Liu et al., 2016; Jia et al., 2017). On the other hand, inhibition of STAT3 is able to suppress cell migration/invasion and significantly downregulate MMP-2 and MMP-9 protein levels in HCC cells (Pei et al., 2016). In this study, we found that EEAC retarded migration and invasion and lowered protein levels of MMP-2 and MMP-9 in cultured cells. These results indicate that EEAC has anti-metastasis potential that should be explored in, and confirmed by, animal models. (25S)-Antcin H is probably one of the anti-metastatic components of EEAC. Noncytotoxic Antcin H has been reported to suppress the invassive capibility of renal cancer cells (Chiu et al., 2017). Further studies are needed to determine the active components responsible for the anit-proliferative and anti-metastatic effects of EEAC.

## Conclusion

In summary, our studies demonstrate that EEAC has anti-HCC effects in cellular and animal models, and suppression of STAT3 signaling contributes to the mechanisms of these effects. Safety assessments indicate that EEAC has no observable toxicity in rats and tumor-bearing mice. Our findings suggest that EEAC has a potential to be developed as an alternative agent for HCC prevention and/or treatment.

## Author Contributions

P-LZ and Z-LY conceived and designed the experiments. P-LZ performed the majority of the experiments, analyzed the data, and drafted the manuscript. Z-LY reviewed the original data and finalized the manuscript. X-QF, J-KL, and AT helped in the animal experiments. HG, C-LY, Y-PW, J-YC, Y-JC, Y-XL, and MH participated in several experiments. All authors read and approved the final manuscript.

## Conflict of Interest Statement

Z-JZ was employed by company Shenzhen Union Assets Biological Technology Co., Ltd. The remaining authors declare that the research was conducted in the absence of any commercial or financial relationships that could be construed as a potential conflict of interest.
