# Supplementary material for: Antrodia camphorata Mycelia Exert Anti-liver Cancer Effects and Inhibit STAT3 Signaling in vitro and in vivo
Source: Front Pharmacol. 2018 Dec 17;9:1449. doi: 10.3389/fphar.2018.01449 (PMC6304454; doi:10.3389/fphar.2018.01449)
Supplement: Supplementary file 1 [file Presentation_1.pdf]

Supplementary material

Antrodia camphorate mycelia exert anti-liver cancer effects and inhibit STAT3 signaling *in vitro* and *in vivo*

Pei-Li Zhu[1,2,3], Xiu-Qiong Fu[1,2,3], Jun-Kui Li[1,2,3], Anfernee Kai-Wing Tse[2], Hui-Guo[1,2,3], Cheng-Le Yin[1,2,3], Ji-Yao Chou[1,2,3], Ya-Ping Wang[1,2,3], Yu-Xi Liu[1,2,3],Ying-Jie Chen[1,2,3], Muhammad Jahangir Hossen[1], Yi Zhang[2], Si-Yuan Pan[3], Zong-Jie Zhao[4], Zhi-Ling Yu[1,2,3*]

***Correspondence:** Zhi-Ling Yu; Email: zlyu@hkbu.edu.hk

Supplementary methods

Determination of Heavy Metals in AC Mycelia

Contents of heavy metals in AC mycelia were detected according to National Standard of the People's Republic of China for determination of Cd, Pb, As and Hg in foods. Briefly, each sample was digested according to the procedures listed in Supporting Table 1. After digestion, transferred the solution to a volumetric flask and diluted to the mark with deionized water. Mixed thoroughly, stored for later use. Analytical reagent blanks and standard series of each heavy metal were also prepared, respectively. Contents of Cd and Pb were determined by graphite furnace atomic absorption spectrometry. Contents of As and Hg were detected by hydride atomic fluorescence spectrophotometry and atomic fluorescence spectrometry, respectively. Cd and Hg were not detected in these mycelia. Contents of Pb and As were estimated to be 0.19 and 0.057 mg/kg, respectively. Contents of these four heavy metals in the mycelia were within the National Standard of the People's Republic of China for Maximum Levels for Contaminants in Foods (GB 2762-2012).

Determination of Aflatoxins in AC Mycelia

Contents of aflatoxins were determined according to GB/T 18979-2003 (Determination of aflatoxins content in food—Cleanup by immunoaffinity chromatography and determination by high-performance liquid chromatography and fluorimeter). Briefly, weighted accurately 25.0 g (m) of the blended sample, added 5 g of sodium chloride and a mixture of methanol and water (7:3), v/v) to 125 mL. Homogenized the mixture for about 2 min after which the solution obtained was filtered using Whatman No. 1 filter paper. Transferred accurately 15 mL of the filtrate to another bottle and then diluted with 30 mL H_2O. The resultant solution was then filtered through a glass-fibre filter by suction. Collect the filtrate as test solution. The test solution was cleaned up by an immunoaffinity column containing antibodies

specific for aflatoxins B_1, B_2, G_1 and G_2. Passed 15 mL of the test solution through the column at a flow rate of about 6 mL/min. Wash the column twice with 10 mL H_2O, followed by passing 2-3 mL of air. Next, 1.0 mL methanol was used as the eluting solution. Elute the column with 1.0 mL methanol at a flow rate of 1-2 mL/min. Collect all eluates. Transferred accurately 1.0 mL of elutes in a 2-mL volumetric flask and made up to the mark with distilled water for assay (diluted elutes). The concentrations of aflatoxins were determined by HPLC analysis. The chromatographic procedure was carried out using a mixture of methanol and distilled water (45+55) as the mobile phase and a flow rate of 0.8 mL/min. 0.05% iodine solution as post-column derivatization reagent, flow rate at 0.2 mL/min, reaction temperature at 70 ℃ and reaction time at 1 min were set as a post-column reactor system. 100 µL of a mixture of aflatoxins standard solution (benzene and acetonitrile 98:2, v/v) or diluted elutes were analyzed by HPLC. Water was used as control. Aflatoxin B1, B2, G1 and G2 were not detected in these mycelia.

Acute oral Toxicity Study

In acute oral toxicity study, 24 rats were randomly divided into two groups, 12 for each (6 females and 6 males). Rats were intragastrically administrated with a single dose of 5000 mg/kg body weight EEAC on day 1 and then observed for next 14 days. The signs of toxic effects and/or mortality were observed carefully every 1 h after administration on the first day, followed by daily observation. Body weights were recorded every week.

Repeated Dose 28-day Oral Toxicity Study

In repeated dose 28-day oral toxicity study, forty SD rats were randomly divided into four groups containing 10 rats for each (5 females and 5 males): control group, 250 mg/kg group, 500 mg/kg group and 1000 mg/kg group. And then EEAC was administrated to rats by gavage once a day for 30 consecutive days.

Hematological and Biochemical Analyses

At the end of the toxicity studies, blood samples were obtained after anesthesia with chloral hydrate. Hematological analysis was performed using an automatic hematological analyzer (Coulter STKS, Beckman). Parameters, including white blood cells (WBC), lymphocytes, monocytes, granulocyte, red blood cells (RBC), hemoglobin and platelets count (PLT), were recorded. For biochemical analysis, following parameters, aspartate aminotransferase (AST), alanine aminotransferase (ALT), total protein, albumin, blood urea nitrogen (BUN), creatinine (CRE), glucose (GLU), total cholesterol (TC) and triglyceride (TG), were determined.

Measurement of Organ Indexes

SD Rats were sacrificed after blood sample were obtained and then gross observation was performed to analyze the macroscopic external features of organs, including heart, liver, spleen, lung, kidney, testis, ovary and uterus. These organs were carefully removed and then weighted individually. Organ index was calculated as follows: (organ weight/ body weight) ×100.

Supplementary data

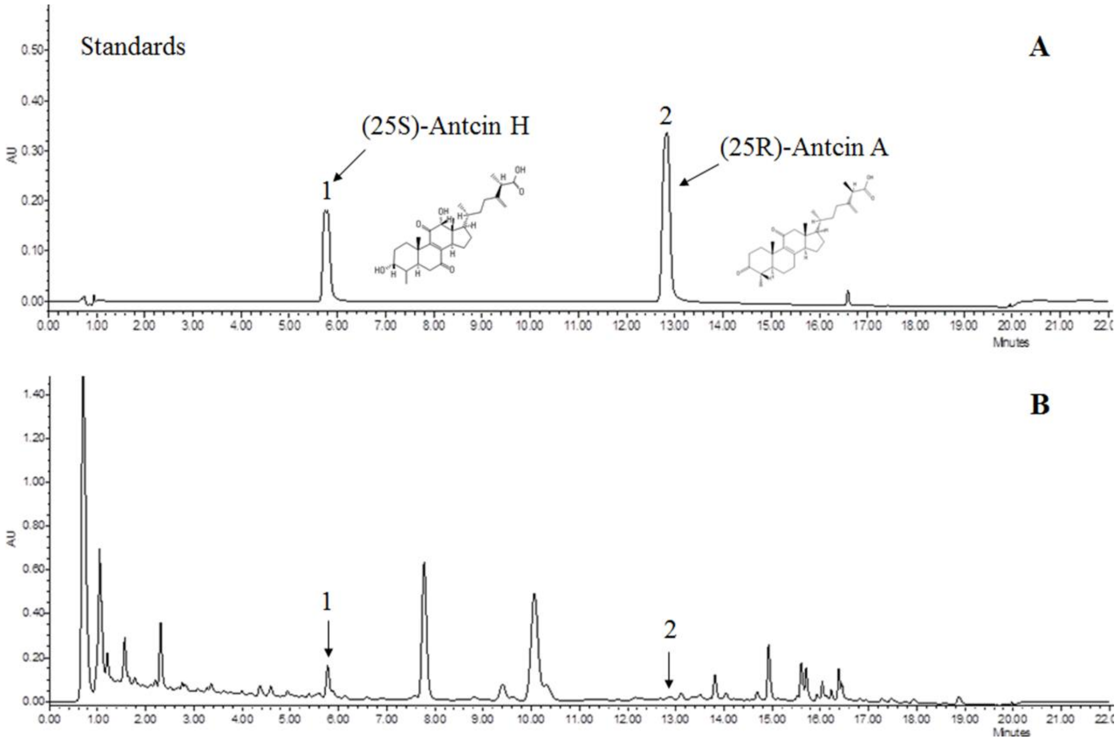

Supplementary Figure S1 UPLC analyses of standards and EEAC. (A) standards: (25S)-Antcin H and (25R)-Antcin A; **(B)** EEAC. UPLC analyses were performed to control the quality of EEAC by using an Acquity UPLC system (Waters Corp., Milford, USA) coupled with HSS T3 column (1.8 μm, 2.1 mm× 100 mm). The mobile phase consisted of A (water containing 0.1% formic acid), and B (acetonitrile). A gradient elution of 30% B at 0-3 min, 58% B at 3-10 min, 70% B at 10-12 min, 95% B at 12-15 min and 95% B at 15-18 min was employed. The column temperature was maintained at 25°C. The flow rate was 0.3 ml/ min, the injection volume was 2 μl and the detection wavelength was 250 nm. UPLC analyses showed that contents of (25S)-Antcin H and (25R)-Antcin A, triterpenoids occurring in AC, in EEAC are 3.87% and 0.08%, respectively.

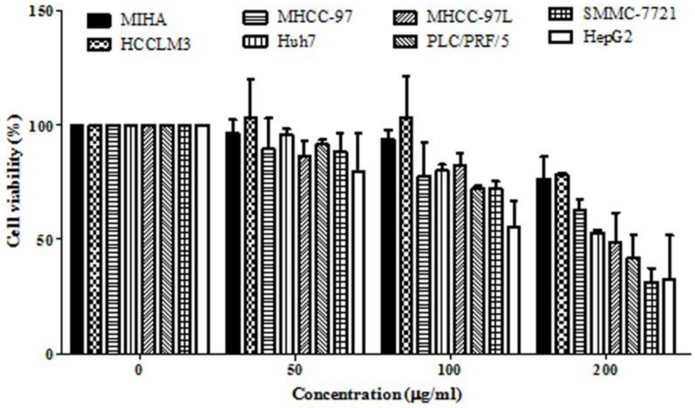

Supplementary Figure S2 Cytotoxic effects of EEAC on human HCC cells and normal human liver-derived (MIHA) cells. Human HCC cells and MIHA cells were treated with various concentrations of EEAC for 24 h and cell viability was measured by the MTT assay. Data were shown as mean ± SD from three independent experiments.

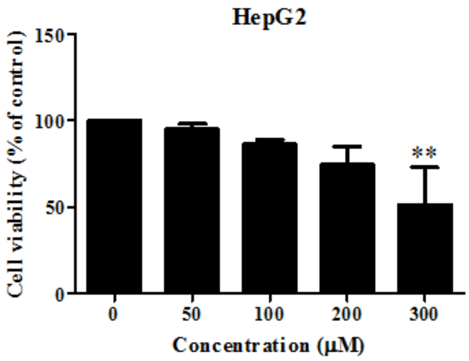

Supplementary Figure S3 Effects of (25S)-Antcin H on the viability of HepG2 cells. HepG2 cell were treated with indicated concentrations of (25S)-Antcin H for 48 h. Cell viability was assessed by MTT assays. Data were shown as mean ± SD from three independent experiments. **P<0.01 vs. the control.

Supplementary Table S1 Digestion procedures for four heavy metals in AC mycelia.

Heavy metal	Reference	Weight sample →	Digest solution →	Soaked →	Added Solution →	Maintained →	Cooled
Cd	GB 5009.15-2014	1 ~ 2 g	HNO_3 5 mL	Overnight	H_2O_2 2 ~ 3 mL	120~160 ℃, 4~6 h	Naturally
As	GB/T 5009.11-2003, method one	1 ~ 2.5 g	HNO_3 20 ~ 40 mL and H_2SO_4 1.25 mL	Overnight	$HCLO_4$ 1 ~ 2 mL	-	Naturally
Pb	GB 5009.12-2010, method one	1 ~ 2 g	HNO_3 2 ~ 4 mL	Overnight	H_2O_2 2 ~ 3 mL	120 ~ 140 ℃, 3 ~ 4 h	Naturally
Hg	GB/T 5009.17-2003, method one	0.2 ~ 1 g	HNO_3 5 mL	Overnight	H_2O_2 **7** mL	120 ℃, 2 ~ 3 h	Naturally

Supplementary Table S2 Body weight (BW) change of rats in acute oral toxicity study ($\bar{x}$ ± SEM, n = 6)

Gender	Dose mg/kg·BW	BW on day 1 (g)	BW on day 7 (g)	BW on day 14 (g)	Toxic sign	Mortality
♀	0	175.8±4.74	188.2±4.94	197.0±5.59	0	0
♀	5000	188.0±3.27	200.5±4.09	208.9±3.20	0	0
♂	0	277.2±4.81	302.7±4.66	325.0±3.46	0	0
♂	5000	278.8±4.87	310.8±5.33	328.5±5.81	0	0

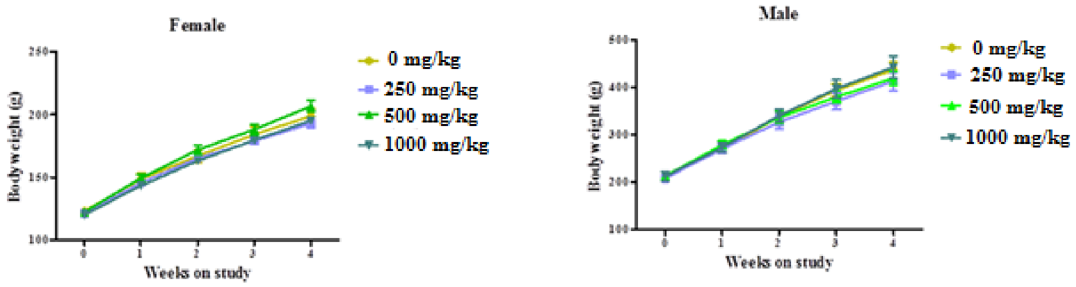

Supplementary Figure S4 Body weights of rats in repeated dose 28-day oral toxicity study. Body weight of rats were recorded every week. Data were presented as mean ± SEM, n = 5.

Supplementary Table S3 Hematological parameters of rats in repeated dose 28-day oral toxicity study ($\bar{x}$ ± SEM, n = 5)

Parameters	Female (mg/kg)				Male (mg/kg)			
	0	250	500	1000	0	250	500	1000
WBC ($\times 10^9$/L)	8.73±1.03	11.43±1.11	11.12±0.73	11.02±0.93	8.98±0.65	10.32±0.95	10.22±0.76	11.80±1.57
Lymphocytes (%)	83.12±0.43	82.10±0.82	81.38±1.48	84.22±0.93	81.92±1.11	83.46±0.88	82.10±1.13	80.18±0.71
Monocytes (%)	2.24±0.05	2.58±0.17	2.42±0.01	2.44±0.09	2.22±0.15	2.34±0.12	2.44±0.18	2.54±0.14
Granulocyte (%)	14.64±0.41	15.32±0.71	16.20±0.61	13.34±0.40	15.86±0.99	14.20±0.76	15.46±1.00	17.28±0.68
RBC ($\times 10^{12}$/L)	5.87±0.68	8.48±1.17	7.82±0.54	9.93±0.73**	6.96±0.25	8.10±0.56	7.11±0.19	7.02±0.18
Hemoglobin (g/L)	110.2±13.9	166.2±23.5	152.6±12.2	193.0±15.8**	142.0±4.29	162.6±11.23	145.4±5.09	144±4.85
PLT ($\times 10^9$/L)	907.6±98.1	1074.4±51.4	886.4±27.72	1126.4±102.2	1078.0±61.1	1124.8±68.1	895.2±74.96*	953.2±66.5

Supplementary Table S4 Serum Biochemical parameters of rats in repeated dose 28-day oral toxicity study ($\overline{x}$ ± SEM, n = 5)

Parameters	Female (mg/kg)				Male (mg/kg)			
	0	250	500	1000	0	250	500	1000
ALT (IU/L)	10.19±0.88	9.78±0.9	10.44±0.64	10.36±1.51	11.43±2.6	9.09±0.51	9.41±0.39	9.57±1.3
AST (IU/L)	20.64±2.41	12.55±0.96*	15.76±0.78	15.30±0.55	18.13±1.23	13.47±1.25*	18.37±1.63	15.93±1.57
Total protein (g/L)	23.98±1.25	23.36±3.04	23.53±2.25	23.48±1.1	23.48±1.47	20.64±0.9	23.87±3.8	21.62±1.27
Albumin (g/L)	3.35±0.1	3.63±0.02	3.36±0.18	3.34±0.07	3.6±0.12	3.97±0.14	3.84±0.12	3.91±0.25
BUN (mmol/L)	9.45±0.15	8.3±0.49	8.82±0.23	9.19±0.32	11.9±0.7	11.24±1.28	11.35±0.55	10.95±0.42
CRE (μmol/L)	69.77±9.38	61.70±14.87	60.35±3.25	68.33±10.83	75.25±5.42	70.62±6.43	82.01±10.64	79.82±5.88
GLU (mmol/L)	19.56±2.31	17.25±2.73	19.49±1.85	23.29±2.39	12.16±0.39	11.36±0.63	12.89±0.73	12.61±0.89
TC (mmol/L)	3.48±1.24	3.64±0.71	2.38±0.17	2.78±0.37	2.98±0.2	3.28±0.233	2.81±0.14	3.26±0.37
TG (mmol/L)	0.32±0.03	0.38±0.02	0.40±0.02	0.39±0.03	0.76±0.1	0.67±0.11	0.86±0.2	0.94±0.09

Supplementary Table S5 Organ indexes (%) of rats in repeated dose 28-day oral toxicity study ($\bar{x}$ ± SEM, n = 5)

Groups	Dose (mg/kg)	Heart (%)	Liver (%)	Spleen (%)	Lung (%)	Kidney (%)	Testis (%)	Ovary (%)	Uterus (%)
Female	0	0.40±0.02	3.70±0.10	0.17±0.01	0.61±0.01	0.86±0.02	/	0.06±0.01	0.18±0.02
	250	0.39±0.03	3.78±0.06	0.18±0.01	0.56±0.03	0.87±0.01	/	0.07±0.00	0.19±0.01
	500	0.40±0.02	3.70±0.07	0.19±0.00	0.57±0.05	0.85±0.02	/	0.06±0.01	0.21±0.01
	1000	0.38±0.01	3.72±0.05	0.18±0.01	0.53±0.02	0.88±0.02	/	0.05±0.00	0.18±0.01
Male	0	0.35±0.01	3.82±0.12	0.19±0.02	0.37±0.02	0.73±0.01	1.00±0.01	/	/
	250	0.36±0.01	3.84±0.06	0.19±0.01	0.39±0.01	0.76±0.01	1.01±0.01	/	/
	500	0.33±0.01	3.79±0.10	0.19±0.01	0.39±0.02	0.75±0.02	1.02±0.01	/	/
	1000	0.33±0.01	3.95±0.13	0.19±0.01	0.38±0.01	0.75±0.01	1.01±0.04	/	/
